# Combined Protein, Probiotics, and Exercise Therapy for Sarcopenia: A Comprehensive Review

**DOI:** 10.3390/cells14171375

**Published:** 2025-09-03

**Authors:** Ryuk Jun Kwon, Mohammad Al Mijan, Soo Min Son, Wanho Yoo, Taehwa Kim

**Affiliations:** 1 Research Institute for Convergence of Biomedical Science and Technology, Pusan National University Yangsan Hospital, Yangsan 50612, Republic of Korea; brain6@hanmail.net (R.J.K.); mijabau@gmail.com (M.A.M.); soo890624@naver.com (S.M.S.); 2 Family Medicine Clinic, Pusan National University Yangsan Hospital, Yangsan 50612, Republic of Korea; 3 Department of Family Medicine, School of Medicine, Pusan National University, Yangsan 50612, Republic of Korea; 4 Biomedical Research Institute, Pusan National University Hospital, Busan 49241, Republic of Korea; 5 Division of Respiratory and Critical Care Medicine, Department of Internal Medicine, Pusan National University Hospital, Busan 49241, Republic of Korea; 6 Department of Internal Medicine, School of Medicine, Pusan National University, Yangsan 50612, Republic of Korea

**Keywords:** sarcopenia, muscle health, protein, probiotics, multimodal exercise

## Abstract

Sarcopenia, a progressive loss of muscle mass and strength, is a major health concern primarily affecting older adults worldwide. With no pharmaceutical cure for sarcopenia, dietary protein, probiotic supplementation, and physical exercise have gained increasing attention as lifestyle-based interventions. Dietary protein has shown promising effects in preventing the loss of skeletal muscle and physical strength by favorably influencing muscle protein synthesis in sarcopenic individuals. Probiotic supplementation has been associated with muscle regeneration, increased muscle protein synthesis among adults with sarcopenia, and improved exercise performance based on preliminary and emerging evidence. Multimodal or hybrid exercise programs have been shown to improve muscle strength, mobility, and overall physical function in individuals with sarcopenia. This paper reviews how combining protein, probiotics, and multimodal exercise may offer complementary strategies for sarcopenia management. Evidence from preclinical and mechanistic studies suggests that these interventions may support muscle health by activating shared intracellular pathways such as mTOR signaling, the suppression of FOXO3a, and the enhancement of mitochondrial biogenesis.

## 1. Introduction

Sarcopenia is a progressive and generalized skeletal muscle disorder associated with an increased likelihood of adverse outcomes including falls, fractures, physical disability, and mortality, as defined by the European Working Group on Sarcopenia in Older People (EWGSOP2) [1]. Similarly, the Asian Working Group for Sarcopenia (AWGS) defines sarcopenia as an age-related loss of skeletal muscle mass plus a loss of muscle strength and/or reduced physical performance [2]. These definitions emphasize the importance of evaluating not only muscle quantity, but also muscle strength and function, when diagnosing sarcopenia [3,4,5]. Based on these criteria, sarcopenia is now widely recognized as a major contributor to the decline in quality of life and adverse health outcomes among older adults [6,7].

**Figure 1:**
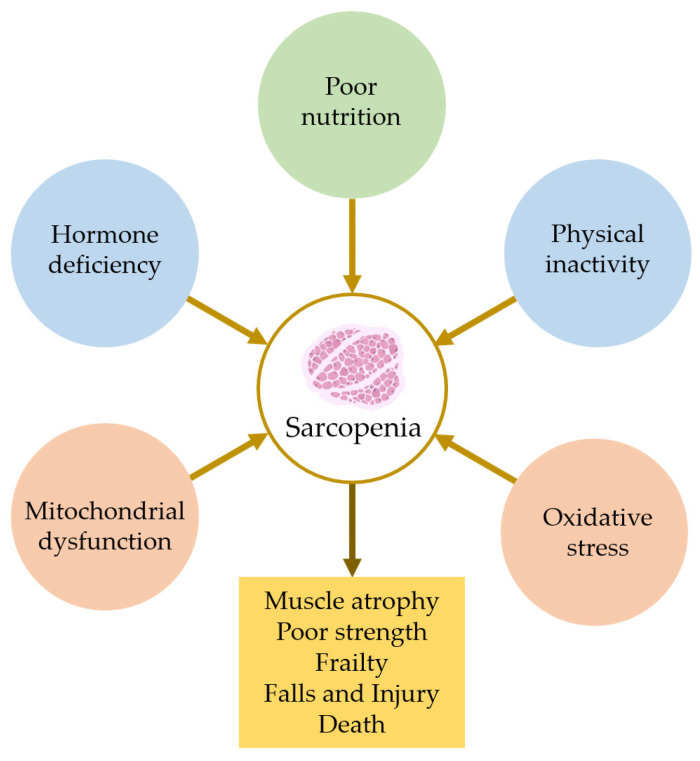
Key risk factors associated with sarcopenia. (Created with BioRender.com).

In the sarcopenic condition, fast-twitch type II fibers gradually convert into slow-twitch type I fibers, accompanied by the infiltration of adipose and connective tissues, weakening the skeletal muscle [8,9]. Multifaceted environmental and genetic factors, including lack of exercise, malnutrition, hormonal imbalance, a decrease in motor neurons, mitochondrial dysfunction, oxidation, and chronic inflammation, are believed to be the primary causes of sarcopenia [10,11] (Figure 1). Despite being one of the predominant diseases in older people, sarcopenia remained unnoticed or was being paid little to no attention until 2016 when International Classification of Diseases, 10th Revision (ICD-10) first recognized it as a disease [12]. Currently, sarcopenia affects between 10 and 16% of the aging population, becoming a massive economic and social burden across the globe [13,14,15].

Many pharmacological agents have been developed to treat sarcopenia, including hormonal therapies, myostatin inhibitors, ACE inhibitors, and nutritional or metabolic supplements [16]. Some of these agents, such as testosterone, xanthine oxidase inhibitors, and bisphosphonates, have progressed to phase-four clinical trials for related conditions [17]. However, no pharmacological treatment has yet received regulatory approval specifically for sarcopenia. As a result, structured physical exercise remains the primary and most effective intervention for sarcopenia management.

Over the last decade, nutritional and lifestyle strategies for sarcopenia have been explored [18,19]. Protein intake provides essential amino acids for muscle synthesis, and leucine-rich protein, in particular, has been associated with improvements in muscle mass and strength [20,21,22,23,24,25]. Probiotics are supported by emerging evidence as potential modulators of muscle metabolism through the gut–muscle axis, although current findings remain preliminary and largely preclinical [26,27,28]. Multimodal exercise, combining aerobic and resistance training, has been shown to improve muscle function and support metabolic and mitochondrial processes [29,30,31]. Sarcopenia has a multifactorial etiology, and single approaches such as protein, probiotics, or exercise alone are often insufficient [18,32,33]. Recent studies indicate that protein supplementation combined with probiotics or exercise can provide synergistic benefits for muscle health in both sarcopenic and healthy adults [34,35,36,37].

The purpose of this review is to synthesize recent evidence on the combined use of dietary protein, probiotic supplementation, and multimodal exercise in sarcopenia, highlighting mechanistic insights and practical implications for future research and individualized interventions. To achieve this, a narrative approach was adopted rather than a systematic methodology, and no time-restricted search was applied.

## 2. Protein Nutrition in Sarcopenia

Muscle loss in sarcopenia reflects a multifactorial process influenced by neurological, hormonal, metabolic, and inflammatory factors [10,11]. In particular, protein degradation often exceeds protein synthesis, which progressively depletes muscle mass and deteriorates quality of life [38,39]. Because of this imbalance, strategies that enhance protein synthesis have been considered critical for mitigating sarcopenia. Adequate dietary protein provides essential amino acids for muscle protein synthesis. According to previous reports, an ample daily protein intake with or without physical exercises resulted in increased skeletal muscle mass and higher physical strength in sarcopenic and in frail individuals [40,41,42,43,44]. Based on clinical and epidemiological observations, older adults and sarcopenia patients may benefit from consuming higher amounts of daily protein than the current general recommendations of 0.8–1.0 g/kg body weight/day, although recommended levels vary across countries and populations [45]. Reportedly, the factors that determine the efficiency of protein supplementation on muscle synthesis in older adults are the protein amount and feeding pattern, synergistic effects of protein with other nutrients, and the quality or the source of protein [33,46]. Moreover, responses to protein supplementation differ among individuals, so personalized nutritional approaches may be needed. Animal proteins are generally considered to be of superior quality in muscle protein synthesis than plant proteins, owing to their well-balanced essential amino acid content, including leucine, lysine, methionine, and valine [47,48]. Leucine and its metabolite, β-hydroxy-β-methylbutyrate (HMB), have been highlighted for their ability to stimulate muscle protein synthesis [18,49,50,51]. A Danish observational study revealed that a concurrent intake of adequate total protein and leucine could help preserve the lean body mass in healthy older adults [52]. Supplementation of leucine-enriched protein could therefore be an effective strategy in preventing muscle protein degeneration in sarcopenic adults.

The benefits of protein supplementation in muscle health could be further increased by combining it with physical exercise and/or probiotics [53]. An earlier study found that both younger and older adults who received protein supplementation during resistance training exhibited greater muscle mass, larger type I and II muscle fiber areas, and higher muscle strength than those who did not [54]. According to Theisen and colleagues, consuming high-quality protein soon after or before physical exercise could be highly efficient in maintaining muscle health [55]. A number of studies reported that the inclusion of probiotics in the diet could enhance the efficiency of protein supplementation in muscle protein synthesis by favorably changing the gut microenvironment for protein metabolism and amino acid absorption [27,56]. Probiotics together with protein supplements have shown benefits in increasing the absorption of leucine and other amino acids, suggesting that co-ingestion of probiotics and protein may contribute to stronger therapeutic actions against sarcopenia although current evidence remains preliminary [27,57]. Taken together, high-quality protein, particularly leucine-rich sources, represents a core nutritional approach for mitigating sarcopenia. The benefits of protein intake may be further amplified when combined with resistance exercise and probiotic supplementation, highlighting the importance of integrated approaches to preserve muscle mass and function.

**Figure 2:**
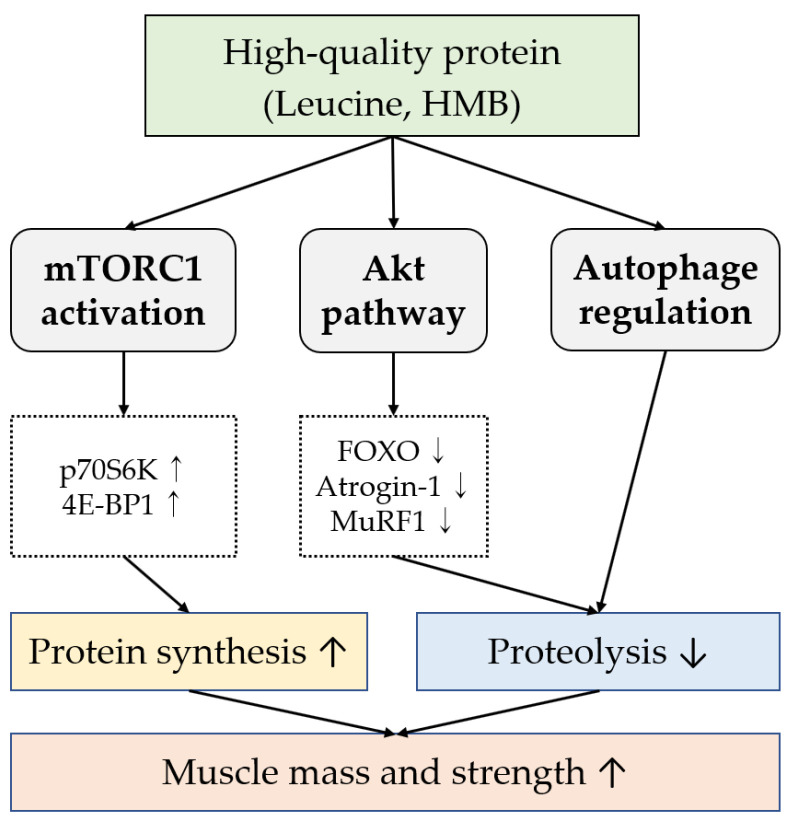
Molecular pathways of dietary protein (leucine, HMB). Arrows indicate activation (↑) or inhibition (↓) of the pathway.

At the molecular level, leucine exerts its anabolic effects primarily through activation of the mechanistic target of rapamycin complex 1 (mTORC1), a central regulator of muscle protein synthesis [23,49,50,51]. Activation of mTORC1 stimulates downstream effectors such as p70S6K and 4E-BP1, which promote translation initiation and elongation of muscle-specific proteins [55]. Concurrently, leucine inhibits muscle protein breakdown by activating the Akt signaling pathway, which in turn suppresses Forkhead box O (FOXO) transcription factors, the key regulators of atrophy-related genes such as Atrogin-1 and MuRF1 [39,49]. HMB has also been shown to attenuate proteolysis and normalize autophagy activity in catabolic conditions by modulating the Akt/FOXO axis [49,51]. In addition, animal proteins such as whey protein provide higher leucine bioavailability compared to plant proteins, contributing to a more robust postprandial stimulation of mTOR signaling in aging muscle, although factors such as digestion rate, amino acid composition, and individual variability may also influence their effectiveness [47,48]. These molecular mechanisms provide compelling support for the role of high-quality protein, particularly leucine-rich sources, as a core strategy in preventing or reversing sarcopenia (Figure 2).

## 3. Microbial Modulation in Sarcopenia

The role of gut microbiota in muscle protein synthesis has drawn considerable attention in recent years, as it modulates nutrient absorption, systemic inflammation, and energy metabolism, all of which are closely related to muscle health [56,58]. In general, older adults with sarcopenia or frailty tend to show reduced microbial diversity and lower populations of beneficial bacteria such as Lactobacilli and Bifidobacteria [18,59]. Previous reports indicate that the dietary supplementation or fecal transplantation of probiotics positively alters the gut microbial population and helps regulate the muscle homeostasis in order to prevent the deterioration of muscle mass and strength [60,61]. Several preclinical studies and small-scale human trials have suggested that altering the gut population with Lactobacillus and Bifidobacteria may help modulate muscle homeostasis and support maintenance of muscle mass and strength [15,60,62]. A systematic review and meta-analysis comprising 24 RCTs recently concluded that the supplementation of probiotic microflora for a long period of time augmented the muscle mass and the global muscle strength in both younger and older adults [62]. The authors suggested that further clarification of the physiological and molecular mechanisms underlying the effects of probiotics on muscle mass and strength in different age groups is needed to guide future research and clinical strategies.

Several studies have suggested that the co-consumption of probiotics and protein may improve muscle mass and physical performance in older adults, although the evidence is still limited [56,63]. Probiotics can support protein digestion and absorption by improving the gut milieu and epithelial transport, thereby increasing amino acid availability and contributing to muscle health [56,64,65]. Recently published observational studies indicate that regular probiotic intake is associated with improved exercise performance and reduced fatigue in athletes [66,67,68]. Although the evidence on the positive role of probiotics in improving physical performance in athletes is gradually increasing, a direct relation between the probiotic supplementation and exercise performance has not been established yet. However, a few observational studies claim that probiotics enhances physical performance in athletes by improving their immune functions and reducing oxidative stress [69]. Taken together, probiotic supplementation may contribute to maintaining muscle homeostasis through effects on protein metabolism and exercise performance, suggesting that the gut microbiota could be a potential therapeutic target in sarcopenia management.

Beyond these clinical observations, recent studies have increasingly focused on elucidating the intracellular molecular actions of probiotics in skeletal muscle. Notably, evidence from preclinical models has shown that probiotic-induced modulation of the gut microbiota can stimulate the IGF-1/PI3K/Akt/mTORC1 signaling pathway, thereby enhancing anabolic processes such as muscle protein synthesis [26,28,62]. At the same time, probiotic supplementation inhibits catabolic signaling through activation of Akt. This pathway suppresses the transcriptional activity of FOXO3a and nuclear factor-κB (NF-κB), which serve as major regulators of muscle protein degradation [28,39,62]. This downregulation leads to reduced expression of atrophy-related genes such as Atrogin-1 and MuRF129. Additionally, specific strains like Lactobacillus paracasei PS23 have demonstrated potential to preserve mitochondrial function and reduce oxidative stress, thereby supporting energy metabolism in aging muscle [15,35]. This is important because aging muscle often shows mitochondrial dysfunction, reduced biogenesis, and lower energy production, which together contribute to the development of sarcopenia [11]. Collectively, these molecular actions reinforce the therapeutic potential of probiotics in combating sarcopenia by modulating the gut–muscle axis and optimizing anabolic–catabolic balance.

## 4. Multimodal Exercise in Sarcopenia

Physical exercise is widely recognized as the mainstay of sarcopenia prevention and management, and the EWGSOP2 consensus highlights it as the first-line and most evidence-based intervention [1,70,71]. Despite being the first-line therapy in counteracting sarcopenia, there is no single exercise program that can be universally recommended to all sarcopenia patients [72]. Both aerobic and resistance exercises have proven benefits in counteracting the challenges of sarcopenia [70,73,74,75]. Nevertheless, given the advantages and limitations of each mode of exercise, a combined exercise training program consisting of both aerobic and resistance exercises is being prioritized in preventing sarcopenia. Gudlaugsson et al. reported that a 6-month multimodal training program in older adults aged 71–90 years, consisting of daily endurance exercise (~30 min at moderate intensity) and twice-weekly resistance training, led to significant improvements in muscle strength, endurance, physical performance, and physical activity [76]. Chen et al. also reported that an 8-week moderate-intensity comprehensive exercise program, consisting of simplified 24-form Tai Chi and progressive resistance training performed three times per week, improved appendicular muscle mass, handgrip strength, gait speed, and chair stand performance in community-dwelling older females with sarcopenia [77]. Recently, a hybrid exercise program combining resistance exercise with Yi Jin Jing, a traditional form of Qigong that emphasizes flexibility, balance, and controlled breathing, was reported to be effective in enhancing skeletal muscle area in older adults with sarcopenia [78]. Hence, rather than using a single exercise plan, devising a multimodal or a hybrid exercise program would be of greater benefit in preventing sarcopenia.

Physical exercise together with protein or probiotics supplementation could provide added benefits to sarcopenia prevention, owing to their individual and synergistic effects in improving muscle health [79,80]. Li and colleagues, in a meta-analysis, found that Asian older adults with sarcopenia experienced increased muscle mass and physical strength with regular exercise and protein intake, indicating synergistic effects between exercise and protein supplementation [81]. In addition, physical exercise can modulate the gut microflora by increasing the number of beneficial bacteria and contributes to the improvement of skeletal muscle health as well as overall health status [79,82]. Therefore, the synergistic effects resulting from the integration of protein, probiotics, and multimodal exercise could offer a powerful solution in counteracting sarcopenia.

Building on these clinical benefits, evidence from preclinical studies has shown that exercise influences multiple intracellular signaling pathways that counteract sarcopenia [39,70]. Physical exercise improves muscle health through multiple molecular pathways involved in protein metabolism, mitochondrial function, and autophagic regulation11 [71]. Resistance training activates the phosphatidylinositol 3-kinase (PI3K)/Akt pathway, which, in turn, stimulates the mammalian target of rapamycin complex 1 (mTORC1), leading to enhanced muscle protein synthesis [55,83]. Concomitantly, exercise downregulates the Forkhead box O3a (FOXO3a) transcription factor and reduces the expression of muscle degradation markers such as Atrogin-1 and MuRF1, thereby preventing muscle wasting [71]. Aerobic exercise has also been shown to induce beneficial adaptations by increasing mitochondrial biogenesis and improving oxidative metabolism through the activation of peroxisome proliferator-activated receptor gamma coactivator 1-alpha (PGC-1α) [73]. In sarcopenic individuals, a combination of aerobic and resistance training has demonstrated synergistic effects by simultaneously improving anabolic signaling and enhancing muscle oxidative capacity [6,70,71]. Furthermore, regular exercise modulates autophagy in aged muscle, restoring proteostasis and attenuating sarcopenia-related cellular damage [11,39]. Taken together, physical exercise, especially multimodal training, appears to exert anti-sarcopenic effects through multiple pathways.

## 5. Integrated Protein, Probiotics, and Multimodal Exercise Therapy in Sarcopenia

**Figure 3:**
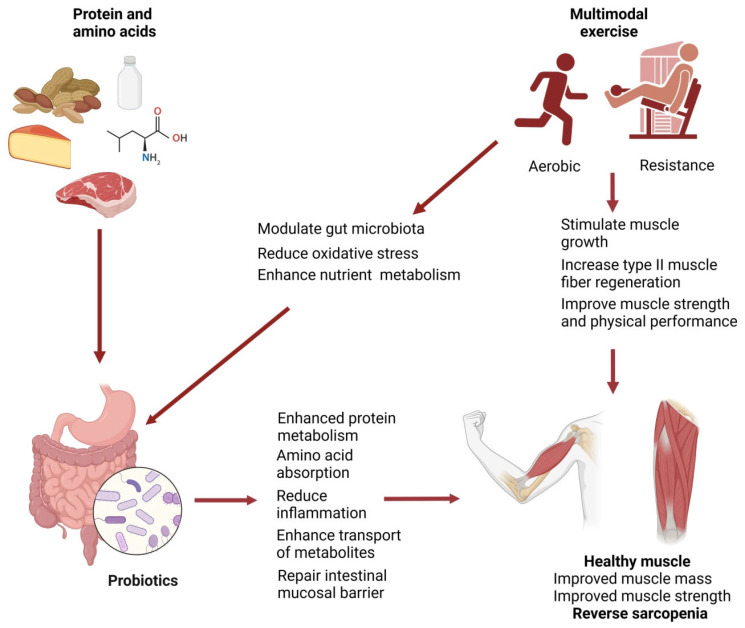
Protein, probiotics, and multimodal exercise as a combined therapy for sarcopenia. (Created with BioRender.com).

The increasing prevalence of sarcopenia along with the inefficiency of the current therapeutic options demands a comprehensive and multidimensional approach. The integration of high-quality protein, probiotics supplementation, and multimodal physical exercise may yield effective solutions to sarcopenia patients, as illustrated in Figure 3, which summarizes the potential synergistic effects of these interventions in enhancing muscle health through multiple physiological pathways. This concept is also consistent with clinical frameworks such as the EWGSOP2 consensus and the recent literature on exercise prescription in older adults, which recommend tailoring exercise programs according to frequency, intensity, time, and type [1,70]. Several clinical trials support the synergistic effects of protein with exercise on muscle mass and strength, with evidence for probiotics still emerging (Table 1).

Protein intake supports muscle regeneration, while probiotics aid in protein breakdown and amino acid absorption as well as dampen inflammatory processes, creating an optimal physiological milieu for muscle maintenance [84,85,86]. Physical exercise complements these efforts by directly stimulating muscle growth and improving overall muscle function [83]. Combined therapies encompassing protein, probiotics, and multimodal exercise have been reported to provide additional benefits compared with single interventions in some studies, although findings remain heterogeneous (Table 1). A new prospective study found positive correlations between a combined therapy of dietary protein, probiotics, and physical exercise and the functional status of the aging population with sarcopenia caused by the prolonged immobilization during the COVID-19 pandemic [87]. This data was corroborated by another study on Korean middle-aged adults, where the subjects attained stronger skeletal muscle and improved physical performance following a combined therapy of whey protein, probiotics, and resistance exercise for 8 weeks [53]. Nucci et al., in their mini review, concluded that the integrated therapies of dietary protein, probiotics, and physical exercise could change the dynamic of the gut–muscle axis and improve muscle health as well as physical performance in all stages of life [88]. A recently conducted clinical trial also suggested that resistance training together with leucine-rich protein intake significantly reversed sarcopenia and frailty in older adults after hospitalization [89].

This figure provides a conceptual illustration of the synergistic effects of combining protein intake, probiotic supplementation, and multimodal exercise in the management of sarcopenia. Dietary protein contributes to muscle health primarily by stimulating muscle protein synthesis. Probiotics complement this effect by enhancing amino acid absorption, reducing inflammation, and improving the gut–muscle axis. Physical activity further supports muscle anabolism by promoting hypertrophy and enhancing mitochondrial function. Together, these interventions form a complementary therapeutic strategy that may promote muscle mass and function in older adults; however, the translation of these mechanistic pathways into consistent and clinically meaningful outcomes remains to be fully established.

**Figure 4:**
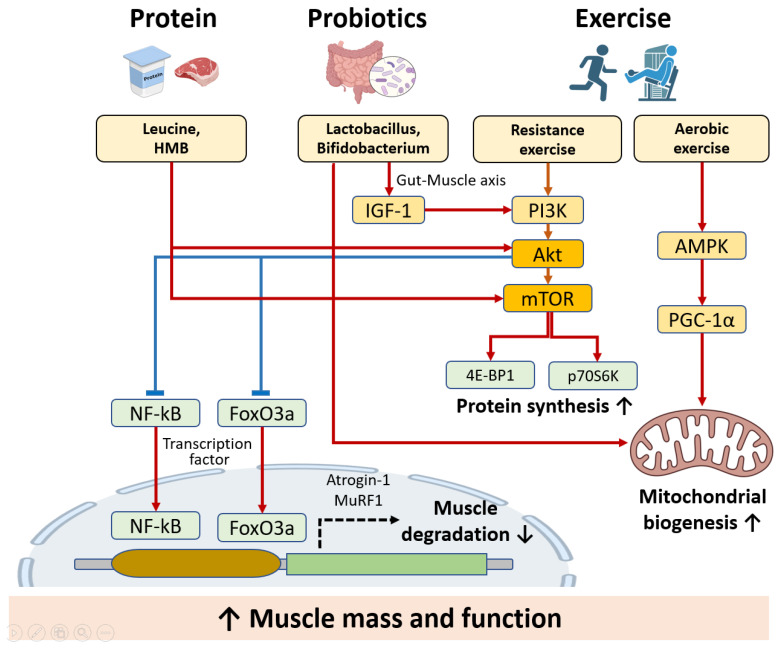
Molecular pathways modulated by protein, amino acids, probiotics, and physical exercise in sarcopenia prevention. (Created with BioRender.com).

To better understand these outcomes mechanistically, Figure 4 illustrates the molecular pathways through which each intervention modulates anabolic and anti-catabolic signaling. Protein, probiotics, and exercise converge on shared targets such as mTORC1 activation, FOXO3a inhibition, and mitochondrial enhancement, ultimately contributing to improved muscle protein synthesis and reduced degradation.

**Table 1:** Summary of results of combined interventions against sarcopenia and muscle aging in older adults.

Type	Population	Intervention	Control	Results	Ref.
RCT	Hospitalized women 60–85 y with knee OA; at risk of sarcopenia	Protein: protein supplementation Exercise: supervised RET Duration: 12 wk	RET alone	↑LMI ↑physical activity	[90]
RCT	26 women ≥60 y with sarcopenic obesity	Protein: whey 35 g/day Exercise: supervised RET Duration: 12 wk	Placebo + supervised RET	↑muscular mass ↑physical strength.	[91]
RCT	49 healthy men ≥ 70 y	Protein: whey-based supplement BID Exercise: exercise program (details NR) Duration: 18 wk	Supplement only	↑muscle mass ↑ muscle strength	[92]
RCT	81 healthy women 65–80 y	Protein: whey 22.3 g post-RET (daily) Exercise: RET Duration: 24 wk	Protein only; exercise only	↑muscle mass ↑gait speed	[93]
RCT	242 adults ≥60 y with sarcopenia	Protein: whey + vitamin D (+fish oil) Exercise: multimodal (AE + RET) Duration: 12 wk	Exercise only; nutrition only; routine consultation	↑muscle mass ↑physical strength	[94]
RCT	31 older women post-THA	Protein: BCAA supplementation Exercise: physical exercise Duration: 4 wk	Placebo + same exercise	↑knee-extension strength ↑upper-limb strength	[95]
RCT	24 adults 60–85 y	Protein: protein 40 g post-RET Exercise: RET Duration: 10 wk	RET alone	↑muscle strength ↑physical performance	[96]
RCT	112 older adults with sarcopenia or dynapenia	Protein: protein + vitamin D Exercise: multimodal (AE + RET) Duration: 12 wk	Exercise only; nutrition only	↑ASM ↑muscle strength	[44]
RCT	165 adults ≥70 y with acute sarcopenia	Protein: whey 27 g/day Exercise: RET 4×/wk Duration: 12 wk	Placebo + same RET	↑skeletal muscle ↑muscle strength	[97]
RCT	200 patients with bed-rest-induced sarcopenia (COVID-19)	Protein: 1.2–1.5 g/kg/day Probiotic: yes (strain/CFU NR) Exercise: physical exercise Duration: 8 wk	No diet/probiotic + same exercise	↑SMI ↑hemoglobin level	[87]

Abbrev.: AE (aerobic exercise); ASM (appendicular skeletal muscle mass); BCAA (branched-chain amino acids); BID (twice daily); CFU (colony-forming units); LMI (lean mass index); OA (osteoarthritis); RET (resistance exercise training); SMI (skeletal muscle index); THA (total hip arthroplasty); post-RET (immediately after an RET session). Symbol: ↑ (increase vs. baseline or control).

Protein and amino acids (including leucine and HMB) activate the mTORC1 pathway, stimulating downstream effectors such as S6K and 4E-BP1 to promote protein synthesis. Additionally, leucine and HMB activate the Akt signaling pathway, leading to the suppression of FOXO3a and NF-κB, which, in turn, reduces the expression of muscle atrophy-related genes (Atrogin-1, MuRF1) and inhibits proteolysis. Probiotics modulate the Akt signaling pathway through the gut–muscle axis, activating IGF-1 and subsequently promoting Akt activation. This action also suppresses FOXO3a and NF-κB, helping preserve muscle mass and function by reducing muscle protein degradation. Additionally, probiotics contribute to mitochondrial biogenesis, further supporting muscle health and energy metabolism. Exercise enhances muscle anabolism by activating the Akt–mTOR signaling pathway, further promoting protein synthesis. Aerobic exercise activates PGC-1α, which improves mitochondrial biogenesis, enhancing muscle function and endurance. These shared intracellular pathways converge to support muscle health and functional performance in older adults.

## 6. Strength and Limitations of Integrated Approaches

Integrated lifestyle strategies for sarcopenia, such as protein supplementation, probiotics, and multimodal exercise, offer several practical advantages. These interventions are not only clinically effective but also relatively cost-efficient compared with emerging pharmacological agents, making them feasible for both clinical and community applications [98,99]. Probiotics are similarly inexpensive and generally safe [100,101], though their long-term clinical benefit remains to be fully established. The overall safety profile of these strategies is favorable, with only mild gastrointestinal discomfort occasionally reported for protein or probiotics, and musculoskeletal strain as a potential risk [79,102,103]. Recent technological innovations have also reinforced these strengths. Home-based digital exercise programs supported by mobile applications or virtual reality have demonstrated improvements in strength, functional status, and mobility in older adults with sarcopenia [104,105]. Furthermore, qualitative evidence from long-term tablet-based balance training programs indicates that older adults generally value the flexibility and confidence gains provided by technology-driven home exercise [106]. In addition, recent interventions using wearable monitoring technologies, including smartwatch-assisted walking programs and activity trackers with step-goal setting, have demonstrated improvements in compliance as well as functional outcomes (grip strength, lower-limb performance) and morphological changes (skeletal muscle mass) in older adults with sarcopenia [107,108]. Emerging exercise physiology research has also highlighted portable tools and imaging methods, such as handgrip dynamometry for strength and ultrasound for muscle thickness, as feasible options to monitor sarcopenia-related changes in clinical and community settings [109,110]. Taken together, these digital health interventions highlight how integrated lifestyle approaches can be further strengthened by enhancing accessibility, sustainability, and patient engagement.

Despite these strengths, important limitations remain. Most clinical trials on integrated strategies are short in duration and heterogeneous in design, limiting the ability to establish long-term efficacy. Evidence supporting probiotics remains preliminary, largely derived from small-scale clinical studies, with some trials reporting modest benefits while others found no significant effects [101]. Similarly, systematic reviews of protein supplementation have shown inconsistent outcomes, failing to demonstrate clear benefits on muscle mass and yielding mixed results for strength and performance [111]. The effectiveness of combined interventions also varies according to baseline nutritional status, comorbidities, and adherence, which are inconsistently reported across studies, leaving critical gaps that must be addressed before these strategies can be fully integrated into standard practice. In addition, standardized strategies for delivering exercise, protein supplementation, and probiotics in older adults are not yet well established, highlighting the need for future research to develop effective implementation models that are feasible in real-world settings.

## 7. Conclusions

The evidence presented in this review suggests the potential of a combined holistic approach to address sarcopenia, primarily for preventive and therapeutic use. It summarizes evidence on the individual effects of dietary protein, probiotic supplementation, and combined exercise, as well as their possible synergistic roles in supporting muscle health and functionality in older adults. High-quality protein ensures adequate amino acid availability for muscle protein synthesis, while probiotics contribute to gut health, potentially influencing amino acid absorption and immune functions. In addition, the inclusion of leucine or consumption of leucine-rich protein appears particularly important. Concurrently, multimodal exercise interventions can address various aspects of muscle function, promoting strength, flexibility, and overall physical performance by utilizing the benefits of both aerobic and resistance exercises. Importantly, recent evidence has uncovered that these interventions exert their effects through interconnected molecular pathways—including mTOR signaling, FOXO3a suppression, and mitochondrial biogenesis—which collectively enhance muscle anabolism and suppress atrophy. Future research should continue to explore the long-term effects, optimal dosages, and potential synergies of these interventions, particularly through long-term randomized controlled trials examining functional outcomes and adherence in diverse older populations, to refine recommendations and enhance their practical application in real-world settings. In addition, further investigations should account for individual variability, including baseline function, comorbidities, and social support, which can strongly influence effectiveness.

## 8. Discussion Questions

(1) How do probiotics and protein supplementation contribute differently to muscle health in older adults?
(2) What are the potential synergistic effects of combining protein supplementation, probiotics, and multimodal exercise in sarcopenia management?
(3) Why is it important to consider individual factors—such as baseline function, comorbidities, or lifestyle habits—when applying multimodal interventions for sarcopenia?
(4) How might digital health technologies improve access and adherence to integrated interventions for sarcopenia?
(5) What types of clinical studies are needed to translate integrated protein–probiotic–exercise strategies from preclinical findings to routine care?

## Data Availability

No data were used in this article.
